# Meiotic Chromosome Contacts as a Plausible Prelude for Robertsonian Translocations

**DOI:** 10.3390/genes11040386

**Published:** 2020-04-02

**Authors:** Sergey Matveevsky, Oxana Kolomiets, Aleksey Bogdanov, Elena Alpeeva, Irina Bakloushinskaya

**Affiliations:** 1 Vavilov Institute of General Genetics, Russian Academy of Sciences, 119991 Moscow, Russia; olkolomiets@mail.ru; 2 Koltzov Institute of Developmental Biology, Russian Academy of Sciences, 119334 Moscow, Russia; bogdalst@yahoo.com (A.B.); alpeeva_l@mail.ru (E.A.); irina.bakl@gmail.com (I.B.)

**Keywords:** *Ellobius alaicus*, translocation, non-homologous chromosome connections, meiosis, synaptonemal complex

## Abstract

Robertsonian translocations are common chromosomal alterations. Chromosome variability affects human health and natural evolution. Despite the significance of such mutations, no mechanisms explaining the emergence of such translocations have yet been demonstrated. Several models have explored possible changes in interphase nuclei. Evidence for non-homologous chromosomes end joining in meiosis is scarce, and is often limited to uncovering mechanisms in damaged cells only. This study presents a primarily qualitative analysis of contacts of non-homologous chromosomes by short arms, during meiotic prophase I in the mole vole, *Ellobius alaicus,* a species with a variable karyotype, due to Robertsonian translocations. Immunocytochemical staining of spermatocytes demonstrated the presence of four contact types for non-homologous chromosomes in meiotic prophase I: (1) proximity, (2) touching, (3) anchoring/tethering, and (4) fusion. Our results suggest distinct mechanisms for chromosomal interactions in meiosis. Thus, we propose to change the translocation mechanism model from ‘contact first’ to ‘contact first in meiosis’.

## 1. Introduction

Chromosomal stability, number and positioning are essential factors for correct genome functionality and inheritance. At the end of 19th century, Rabl hypothesized the non-random, three-dimensional location of chromosomes in the nucleus, whereas in the last two decades, these observations have been advanced with data from advanced technological methods, including fluorescence in situ hybridization (FISH), immunocytochemistry and others [1,2,3]. Information on the tissue-specific positioning of chromosomes has revealed functional nuclear regulation [4,5], or altered states in cancer cells [6,7]. Chromosomal changes at the individual development are usually highlighted as catastrophic genomic events, while such chromosomal alterations often result in carcinogenesis and infertility [8,9]. Genomes of carcinogenetic cells are highly dynamic, and in some cases, chromosomal changes, either induced or spontaneous, lead to therapeutic resistance [10]. Chromoanagenesis, encompasses chromothripsis, chromoanasynthesis and chromoplexy, and was recently described as a possible mechanism contributing to chromosomal evolution [11,12,13]. Even though a rearranged genome may suffer maladaptive modifications, some variations are beneficial for organisms or species in terms of advantageous natural selection. Karyotypic diversity, structural variations of autosomes and sex chromosomes exemplify the importance of chromosomal changes throughout evolution [14,15,16,17,18,19].

Distinct drivers for chromosomal change have been identified, e.g., mobile Penelope elements implicated in *Drosophila* speciation [20], LINE-1 elements in mammals [21], and noncoding RNAs [22] etc. These factors destabilize genomes, initiate DNA damage, and provoke the abnormal linking of chromosomes. At least two translocation models based on non-random chromosome distribution in the nucleus have been proposed [23].

An initial step in “breakage-first” models are double-strand breaks (DSBs). Then potential partners occasionally tie up and produce changed chromosomes, which can obtain distinct fragments of non-homologous chromosomes, up to the whole arm, e.g., Robertsonian translocations (Rbs) or Whole-Arm Reciprocal Translocations (WARTs) [23]. Translocation probability rates could be higher if chromosomes were located close to each other in the nuclear space [24]. For intermingling chromosomes, DSBs in contact zones can lead to non-homologous linking and translocations [25,26]. In this context, the evolutionary integrative breakage model [27] stressed determining the genomic distribution of evolutionary breakpoints due to particular DNA sequence composition and the nucleome, combined with alteration of gene expression due to genome reshuffling.

In the ‘contact-first’ models, chromosomes should be broken, but DSBs start in colocalized chromatids inside specific protein complexes [28]. Firstly, chromosomes come together, then undergo DSBs and join with other partners. Chromosome region mobility is different during the cell cycle; it is higher in the early G1 phase, but decreases in the S phase. In an interphase nucleus, chromatin status, positioning, and cytoskeleton mechanical forces, all influence chromosome movement [29]. The combined variant of both models was also proposed. If DSBs occur in G1 and are not repaired until G2, they may cluster and form translocations [30].

In all models, the fate of small acrocentric chromosomal arms is uncertain; but most probably, they are eliminated. It is important to highlight these models were developed for interphase nuclei when chromosomes were decondensed and occupied specific chromosomal territories. Moreover, the tissue-specific positioning of chromosomes in interphase nuclei [31], enables the formation of distinctive carcinogenic translocations [8], which better fit the first model.

When we investigate the altered three-dimensional organization of somatic cells with re-arranged chromosomes, we cannot immediately determine the evolutionary consequences. How will such translocations pass to the next generations? Therefore, we must look for genome rearrangements in the germline. De novo chromosome rearrangements can arise during germ cell proliferation, meiosis, and in haploid sperm or eggs [32]. Any rearrangement provoking genomic instability may be beneficial for diversification and genetic speciation. 

A specific fusion between acrocentric chromosomes, ended by metacentric chromosomes was first described by Robertson [33], and later named Robertsonian translocations (Rbs). The frequency of such translocations in humans is high: approximately 1 in 1000 individuals [34]. The most common rob(13q14q) and rob(14q21q) translocations originate during oogenesis [35]. A breakpoint diversity exemplified distinct ways for Rb formation, the significant input of the pre-meiotic replication and proper meiotic recombination [36,37]. Probable mechanisms for the formation of Rbs involving telomere changes were suggested [38]. One of the potential mechanisms may be a loss of p-arm telomeres when chromosome breakage occurs within minor satellite sequences [39,40]; another way is a fusion without any losses, and inactivation of telomeres [41]; the one more way may operate via the deletion/inactivation of the telomerase RNA gene which induces telomere shortening [42]. Data on meiosis are scarce and are limited to non-homologous end joining (NHEJ) in mouse spermatocytes after gamma radiation [43], however telocentric chromosome associations in the pachytene are observed for *Mus domesticus* [44].

The high frequency of translocations in humans and the evolutionary input of Rbs requires exploration of non-model species to reveal origin and maintenance mechanisms. Several mammalian species have demonstrated natural variability’s in chromosomal numbers, including Rbs. *Mus, Sorex, Ellobius* species, and some others exhibit changes in diploid numbers, along with stable fundamental numbers due to whole branch fusions [45,46,47,48]. Recently, using chromosome painting, we described karyotype structures in three cryptic *Ellobius* species, *E. talpinus*, *E. tancrei,* and *E. alaicus*; we demonstrated a homology of re-arranged chromosomes, and showed the existence of XX sex chromosomes in males and females [49,50,51]. We hypothesized that a neocentromere origin in one pair of chromosomes was an initial disturbance event for the *E. tancrei* (2n = 54–30) genome, in contrast to stable *E. talpinus* (2n = 54) [49,52]. *E. alaicus* is very close to *E. tancrei*, a translocation Rb(2.11) emerged in both species; other Rbs are species-specific ones. *E. alaicus* demonstrates rapid chromosomal changes in nature, such as the fixation of Rbs in the large population in the Pamir-Alay [50]. In this study, we analyzed the meiotic sustainability of species with rapidly evolving genome.

## 2. Materials and Methods 

### 2.1. Material and Mitotic Chromosomes

We used samples of 6 specimens of *E. alaicus*, kept in the cytogenetic collection (a part of the Joint collection of wildlife tissues for fundamental, applied and environmental researches of the Koltzov Institute of Developmental Biology RAS, Core Centrum of the Koltzov Institute of Developmental Biology RAS, state registration number 6868145). Two males and two females (collection numbers; 27353, 27357, 27354, 27356) were karyotyped using bone marrow suspensions [53]. Tissue from another male (27532) and female (27351) were also used to derive somatic cell cultures. C-band staining of mitotic metaphase plates was performed according to Sumner [54].

We followed international, national, and institutional guidelines for animal care. Studies were approved by the Ethics Committee for Animal Research of the Koltzov Institute of Developmental Biology RAS and the Vavilov Institute of General Genetics RAS.

### 2.2. Cell Culture 

Chondrocyte and fibroblast cell lines were obtained from the Cell culture collection of the Koltzov Institute of Developmental Biology RAS. Chondrocytes were cryopreserved after the first subcultivation and fibroblasts after the third subcultivation and were stored at −196 °C in liquid nitrogen using cultivation media with 10% DMSO for cryopreservation. Karyotype evaluation was performed after the cells were recovered after freezing.

### 2.3. Meiotic Chromosome Studies and Immunostaining

Samples from three *E. alaicus* (27352, 27353 and 27357) adult males were used for the meiotic study. Synaptonemal complex (SC) preparations were made and fixed according to Peters et al. [55], with some modifications [56].

Primary antibodies used for immunostaining: rabbit anti-synaptonemal complex protein 3 (SYCP3) antibody (diluted 1:250, Abcam, Cambridge, UK); human anti-centromere Calcinosis Raynaud’s phenomenon, Esophageal dysmotility, Sclerodactyly, and Telangiectasia (CREST) antibody (CREST, 1:250, Fitzgerald Industries International, USA); mouse anti-phospho-histone H2AX (diluted 1:250–500, Abcam) (also known as γH2AFX); rabbit anti-H3K9me3 antibody (1:100, Abcam; kindly provided by Dr. Jesus Page). 

As secondary antibodies we used goat anti-rabbit IgG, Alexa Fluor 488-conjugate (Invitrogen, Carlsbad, CA, USA); goat anti-human IgG, Alexa Fluor 546-conjugate (Invitrogen); goat anti-mouse IgG, Alexa Fluor 546-conjugate (Invitrogen, USA) (diluted 1:250–500). Slides were washed in phosphate-buffered saline (PBS) and placed into Vectashield, with 4′,6-diamidino-2-phenylindole (DAPI) (Vector Laboratories, USA). Slides were analyzed using a fluorescence light microscope, Axio Imager D1 (Carl Zeiss, Jena, Germany). Immunostaining was described previously [57,58]. We immunostained H3K9me3 histones using two approaches; 1) the first round of SYCP3 staining, then a second round—H3K9me3 or 2) the first round of H3K9me3, then a second round of SYCP3 staining.

SCs measurements in 41 spermatocytes were performed using the MicroMeasure program (Colorado State University, CO, USA).

## 3. Results

### 3.1. Mitotic Chromosomes and SC Karyotype of E. alaicus

All animals demonstrated normal for *E. alaicus* karyotypes; 2n = 52. the fundamental number of chromosome arms (NF) was 56 (Figure S1a–f), consisting of one pair of submetacentrics (№7), characteristic of *E. tancrei* and *E. alaicus*, and one pair of large Robertsonian metacentrics 2(Rb2.11), typical of *E. alaicus* [50]. Mitotic metaphases from bone marrow suspensions showed no visible alterations (Figure S1b). Mitotic metaphases from fibroblast cultures demonstrated a stable karyotype in one male (Figure S1a), and a small number of deviations in one female, i.e., associations and polyploid cells. In chondrocyte cultures of the same specimens, more associations and polyploid cells were identified in female cells (Figure S1d–f), alongside with a large number of micronuclei, disturbed anaphases and massive chromosomal changes (Figures S1g and S2).

**Figure 1:**
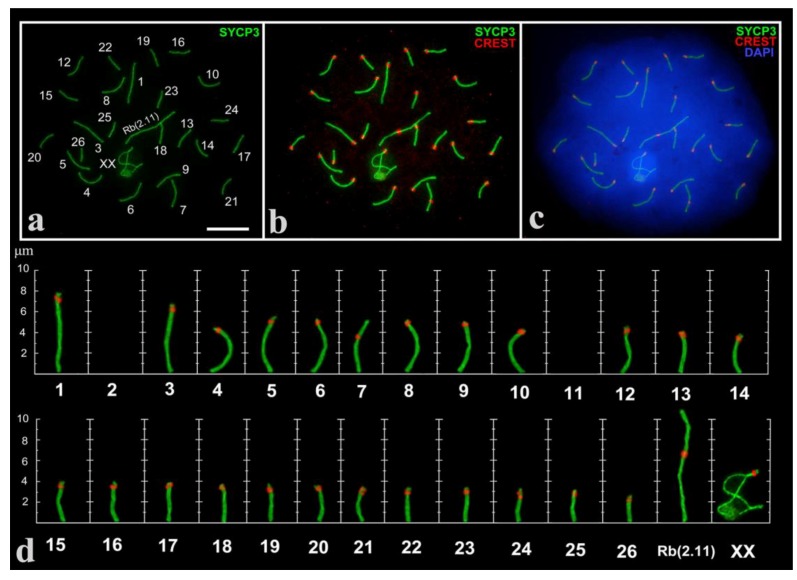
Pachytene spermatocyte (**a**–**c**) and synaptonemal complex (SC) karyotype (**d**) of the Alay mole vole, *E. alaicus*. SCs were immunostained with antibodies against synaptonemal complex protein 3 (SYCP3) (green) and centromeres—with antibodies to kinetochores (Calcinosis Raynaud’s phenomenon, Esophageal dysmotility, Sclerodactyly, and Telangiectasia (CREST), red). The 4′,6-diamidino-2-phenylindole (DAPI)-stained the chromatin (blue). SC numbers corresponded to the metaphase karyotype. Rb(2.11)—Robertsonian submetacentric. Chromosome №7 is non-Robertsonian submetacentric. XX—male sex chromosomes. Bar (**a**–**c**) = 5 µm.

A total of 302 spermatocytes at different prophase I stages in three males were analyzed. As expected, in the pachytene stage we revealed 25 fully synapsed autosomal bivalents (large Robertsonian metacentric Rb(2.11)), one mid-size submetacentric №7 with neocentromere [49,52]; 23 acrocentrics and an XX sex bivalent (Figure 1), formed by two large acrocentrics. Analyzing large cell numbers made it possible to distinguish a range of gradually decreasing in size acrocentrics.

### 3.2. Prophase I Stages in E. alaicus

**Figure 2:**
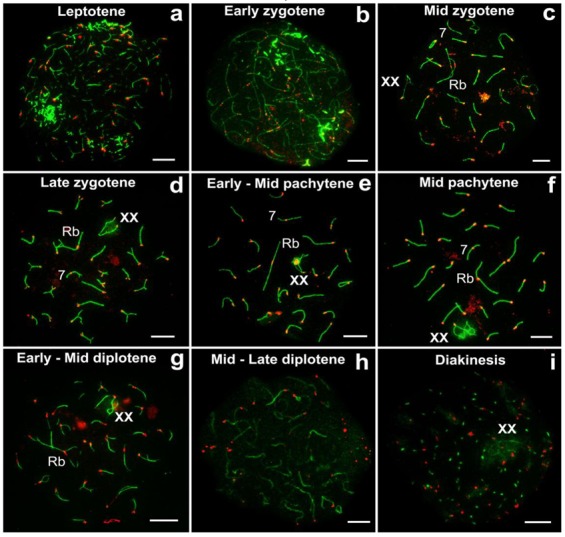
Prophase I stages in *E. alaicus* males. Axial/lateral elements were identified using anti-SYCP3 antibodies (green) and kinetochores (red) using CREST. During the leptotene stage, numerous thin SYCP3 fragments and SYCP3 conglomerates were formed (**a**), formed axial elements (**b**) were visible in the zygotene. Chromosome synapsis began with either distal (telomeric) (**c**) or central chromosome parts (**d**). At the pachytene stage, 25 autosomal SCs and sex (XX) bivalents were formed (**e**,**f**). The XX bivalent demonstrated synapsis at telomeric areas and asynapsis at the central region (**e**,**f**). In the mid-late diplotene, desynapsis progressed from both the central and telomeric areas (**g**). SYCP3 was degraded (**h**) and retained as dots (**i**). Rb—Robertsonian submetacentric. Chromosome 7 is non-Robertsonian submetacentric. Bar (**a**–**i**) = 5 µm.

All prophase I stages were demonstrated for *E. alaicus*. Thin short SYCP3 fragments and SYCP3 conglomerates were formed in the leptotene stage (Figure 2a). In the early zygotene stage, long axial elements were visible, and SYCP3 blocks were kept (Figure 2b). As the zygotene progressed, the axial elements of homologous chromosomes began to contact each other (Figure S3), and synapsed more frequently from telomere areas to the central part (Figure 2c), less often in the opposite way (Figure 2d). In the pachytene stage, chromosomes were completely synapsed (Figure 2f). In the diplotene stage, chromosome desynapsis might be different within a single cell, from telomere sites to the center, or vice versa (Figure 2g). Then SYCP3 degraded (Figure 2h), and it was preserved as dots in the diakinesis stage (Figure 2i).

Sex (XX) chromosomes from early to mid zygotene were detected as separate axial elements (Figure S3). From the mid-late zygote, the XX chromosomes became well visible (Figure 2c). The sex bivalent is similar to those of the cryptic species, *E. talpinus* [59] and *E. tancrei* [60,61]. In the middle pachytene, the XX-bivalent was usually shifted to the periphery of the meiotic nucleus and had two telomeric synaptic regions, a wide asynaptic region, and chromatin bodies on axial elements (Figure 2f). It should be noted that sometimes chromatin (nucleolus-like) bodies in sex bivalents were SYCP3-positive (Figure 1, Figure 2e,f), which has not been previously observed for the other two cryptic species and *E. alaicus* (2n = 48) from the Pamir–Alay [50].

### 3.3. Types of Non-Homologous Acrocentric Connections

**Figure 3:**
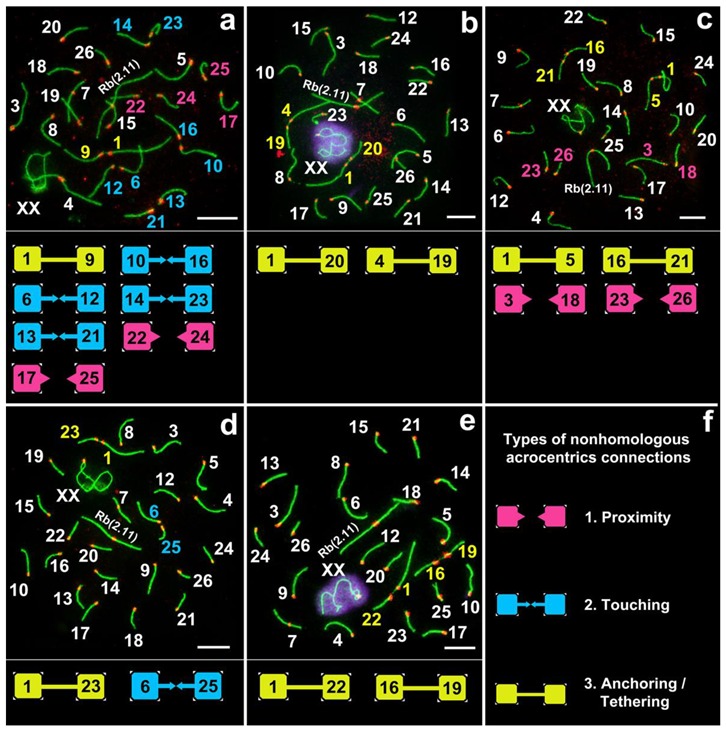
Different chromosome combinations in pachytene spermatocytes of *E. alaicus* (**a**–**f**). Axial elements were identified using anti-SYCP3 antibodies (green), kinetochores using CREST antibodies (red), and anti-γH2AFX (violet) was used as a marker of chromatin inactivation. Pink, blue, and yellow squares correspond to different types of non-homologous acrocentric connections (**f**). Bar (**a**–**e**) = 5 µm.

**Figure 4:**
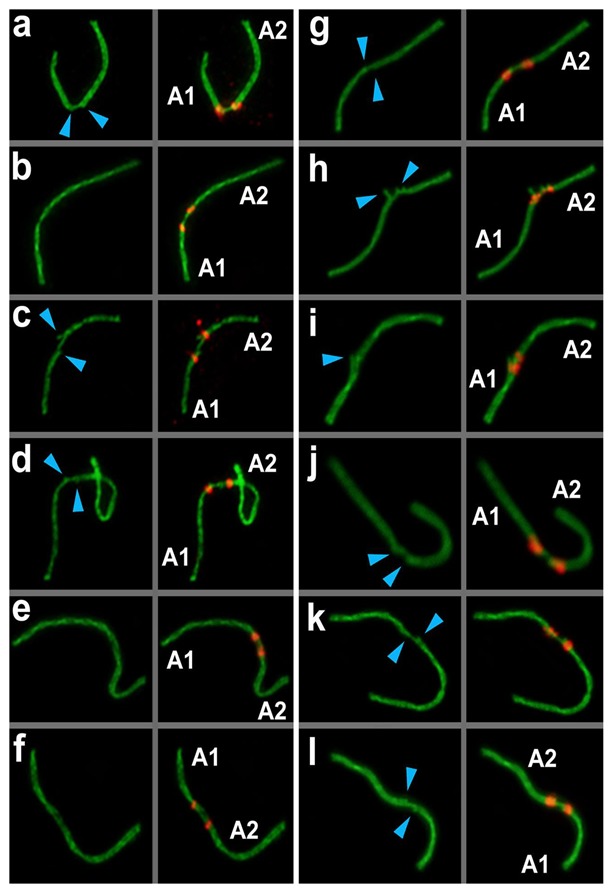
Anchoring/tethering and fusion of non-homologous acrocentric chromosomes in *E. alaicus* spermatocytes (**a**–**l**). Lateral elements were identified using anti-SYCP3 antibodies (green), and CREST antibodies for kinetochores (red). A1 and A2—contacting non-homologous acrocentrics. Blue arrowheads show the free ends of lateral elements of non-homologous acrocentrics.

**Figure 5:**
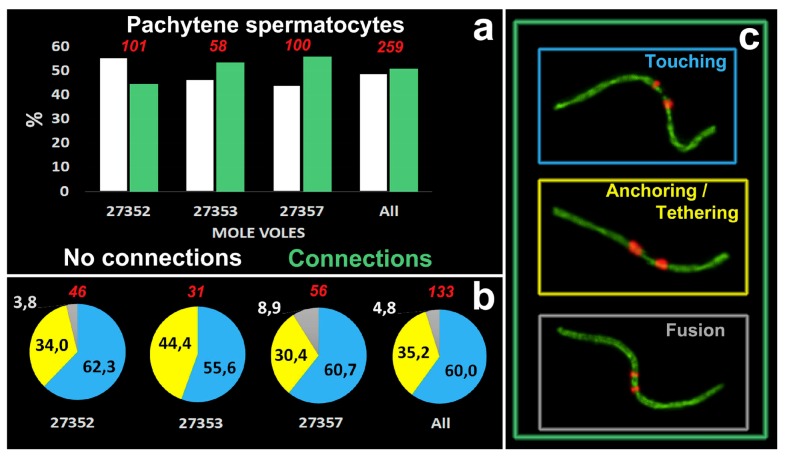
Quantitative characteristics of meiotic contacts in *E. alaicus*. (**a**). The diagram shows the percentage of pachytene cells with or without meiotic connections in three mole vole males (№27352, 27353, 27357). ‘Touching’, ‘anchoring/tethering’ and ‘fusion’ types were counted in cells with connections only. (**b**). Proportions of connection types. Blue color corresponds to ‘touching’, yellow—to ‘anchoring/tethering’ and gray—to ‘’fusion’’. Red numbers are counted pachytene spermatocytes (**a**,**b**). (**c**). Types of contacts that are included in the diagrams. The color of the frames corresponds to the types of contacts (**b**).

**Figure 6:**
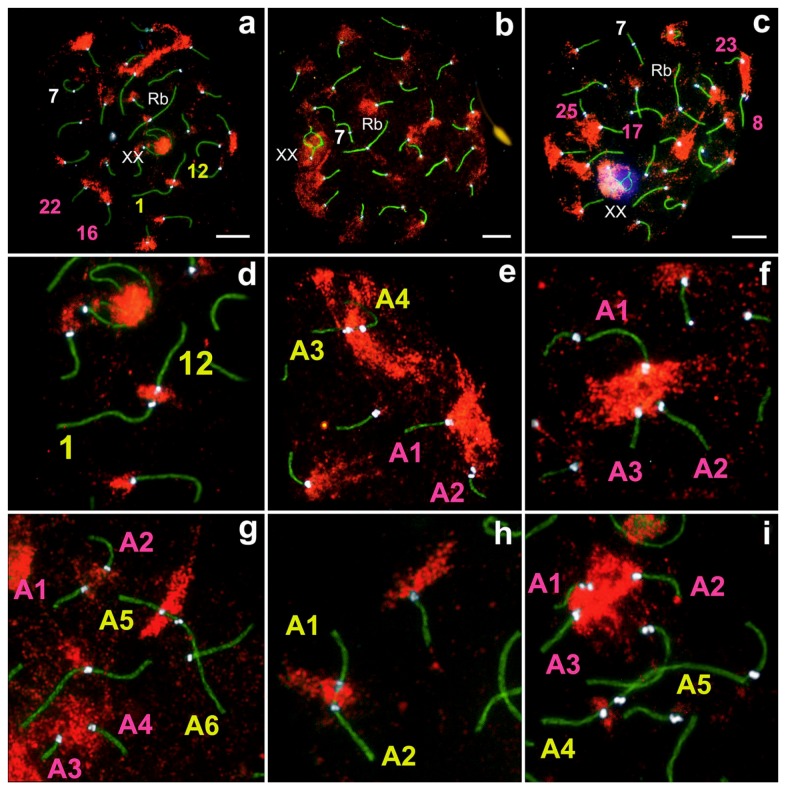
Location of SYCP3 (green), H3K9me3 (red), CREST (white) and gamma-H2AFX (violet) in the pachytene stage of prophase I of *E. alaicus* spermatocytes (**a**–**i**). Size bar = 5 µm (**a**–**c**). (**d**) an enlarged fragment of (**a**), (**e**–**i**) enlarged fragments of distinct cells. Designations A1–A6 do not correspond to numbers of chromosomes as in Figure 1. See the explanation in the text.

**Figure 7:**
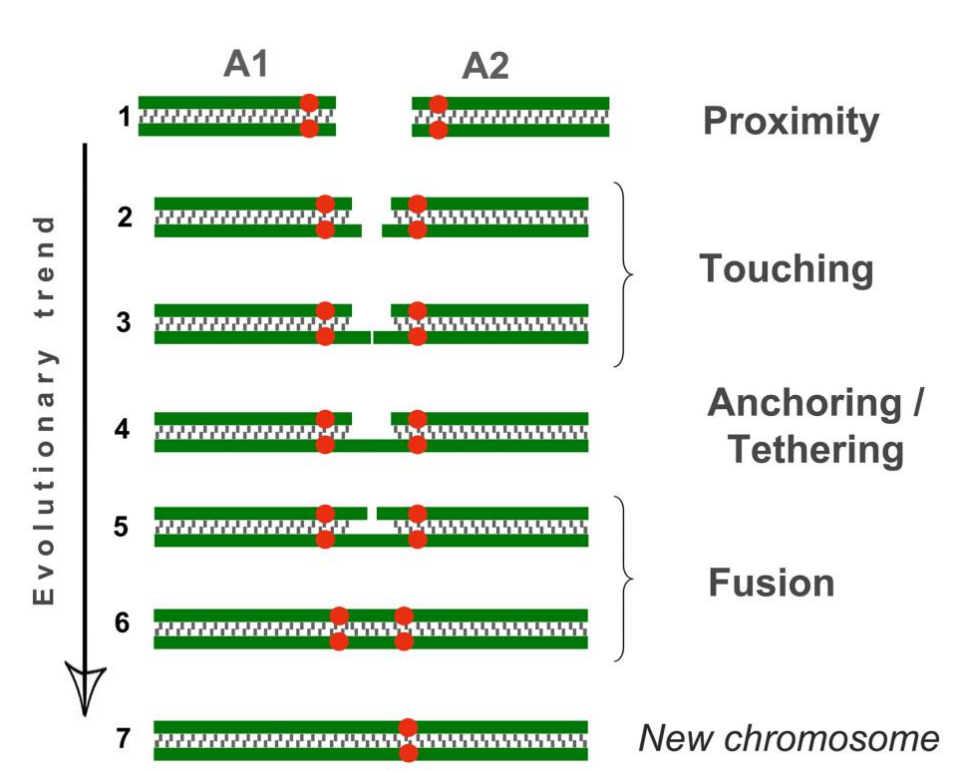
Different types of chromosome connections in meiotic prophase I. The scheme is based on observations of chromosome behaviors during *E. alaicus* meiosis. Green corresponds to SC axial/lateral elements, red dots refer to centromeres. Non-homologous acrocentrics (A1 and A2) occupy positions in the nucleus, intending to get closer (proximity, position 1). One of two lateral elements in the short arm A1 and A2 are extended (position 2), touching each other (position 3) (touching) and eventually bind together (anchoring/tethering, position 4). The other two axial elements are tightened to each other. (Position 5) and probably joined together (position 6) (fusion). After the probable binding of the two axial elements, the centromeres are located somewhat closer to each other (compare positions 5 and 6). Such dicentric bivalents were found in all oocytes of low-chromosomal forms of *E. tancrei,* 2n = 34 (Figure S8). In such dicentric chromosomes, one centromere may be inactivated, with a new chromosome emerging (position 7).

Various non-homologous chromosome contacts were identified in meiotic prophase I, during zygotene-early pachytene stages. We revealed at least four sequential contact and link types of chromosomes (Figure 3, Figure 4, Figure 5, Figure 6, Figure 7).

#### 3.3.1. Proximity

Acrocentrics occupied spatial positions, intending to get their centromeres closer (pink squares and pink numbers of chromosomes in Figure 3, Figure 7, Figures S4 and S5). The distance between the chromosomes was about 1–2 microns. Centromere regions of chromosomes were located around the H3K9me3-domain (Figure 6 and Figures S5–S7). This type precedes true meiotic contacts (see the following types). This ‘proximity’ type was somewhat subjective, because it can easily be confused with closely located chromosomes.

#### 3.3.2. Touching

Acrocentrics moved closer to each other. In each contact acrocentric, one axial element of the short arm was extended, reaching out to one another as if touching each other with their ends (blue squares and blue numbers of chromosomes in Figure 3, Figure 5, Figure 7, Figures S4 and S5). Usually, the distance between the chromosomes is less than 1 micron.

#### 3.3.3. Anchoring/Tethering

One of the axial elements in the short arms of non-homologous acrocentrics were linked to each other by SYCP3-filament (yellow squares and yellow numbers of chromosomes in Figure 3, Figure 4, Figure 5, Figure 7, Figures S4 and S5). Other axial elements of the short arms of the two non-homologous partners were not connected (see blue arrowheads in Figure 4 and Figure 5c).

#### 3.3.4. Fusion

The other two axial elements in the short arm of the non-homologous acrocentrics were tightly adjacent to each other, or possibly connected entirely. The two acrocentrics likely represented a single bivalent with two centromeres (Figure 4e,f, Figure 5c). Centromeric regions were closer to each other compared to other connection types. Such dicentric bivalents were observed in some low-chromosomal forms in *E. tancrei* (Figure 7 and Figure S8).

Such linkage was evidenced by immunostaining; two thin SYCP3-positive filaments (Figure 4e,f). The difference between ’anchoring/tethering’ (see filaments between chromosomes 5 and 6 in Figure S4e) and ‘fusion’ (Figure 4e,f; see chromosomes 14 and 18 in Figure S4e) can be determined by the presence of a complete bridge, with a short distance between two contacting non-homologous chromosomes. In all cases, the contact was made by the short arms of acrocentric chromosomes by SYCP3-filaments (see enlarged fragments of cells; Figure 4).

51% of pachytene cells had meiotic contacts including ‘touching’, ‘anchoring/tethering’ and ‘fusion’ types and excluding ‘proximity’ (Figure 5a). The patterns of proportion of different connection types were similar in three males: the number of ‘touching’ was greater than the number of ‘anchoring/tethering’, the number of ‘fusion’ was less than two other types (Figure 5b). The ‘fusion’ type was not found in one male, №27353 (Figure 5b). The rarity of ‘fusion’ type may be due to the fact that more molecular events should precede the joining/fusion of the axial elements of the short arms of two non-homologous acrocentrics.

Chromosome combinations in pachytene spermatocytes of *E. alaicus* were numerous. We were unable to determine the trend in the frequencies of contact between certain chromosomes. However, chromosomes 1,4,5,20–22 came into contact more often. For example, chromosome №1, which was the most regularly seen in all three types of true interactions (‘touching’, ‘anchoring/tethering’, and ‘fusion’). Rb(1.3) was described in *E. alaicus* with 2n = 50 [50].

### 3.4. Histone H3K9me3 in Prophase I and Meiotic Chromosomes Contacts 

We investigated H3K9me3 (trimethylation of H3 lysine 9) immunolocalization and distribution in contacting chromosomes. H3K9me3 is an epigenetic marker for heterochromatin allocation in prophase I [62].

During the zygotene stage, large clouds of H3K9me3 were localized at pericentromeric regions of axial elements, or at fully formed SCs (Figure S6a–c). In the pachytene stage, clouds of H3K9me3 were reduced in size and were clearly localized to SC pericentromeric regions (Figure 6a–c, Figure S6d–i). Rb-metacentrics had minimal H3K9me3 levels in the centromeric region (Figure 6a–c, Figure S6d–i), or were absent. Non-Robertsonian submetacentric №7 did not demonstrate clear H3K9me3 signals in all studied cells (Figures S6e–h and S7a–c). H3K9me3 usually shrouded one of the axial elements, and a chromatin body inside the sex bivalent (XX) (Figure 6a–d); less often H3K9me3 covered it entirely (Figure S5g,h), whilst γH2AFX totally enclosed XX (Figure 6c and Figure 3b,e). A more detailed description of XX sex chromosomes in *E. alaicus* will be discussed in future work.

In non-homologous acrocentric contacts, H3K9me3 was involved in varying volumes, usually in centromeric regions. We detected a large H3K9me3 cloud between chromosomes at the ‘proximity’ type (pinks points in Figure 6a,c,e–g,i). For closer chromosome contacts, i.e., ‘touching’ and ‘anchoring/tethering’, H3K9me3 distribution ranged from average to insignificant (small) (yellow points in Figure 6a,d,e,g,i). If contacts involved shorter and longer acrocentrics, H3K9me3 signals were located closer to centromeric regions of shorter acrocentrics (Figure 6g,i). For other cases, H3K9me3 signals were clearly identified between the two centromeres of contacting chromosomes (Figure 6d,h).

### 3.5. Mitotic Cells

In contrast to meiotic cells demonstrating ‘touching’, ‘anchoring’, and ‘fusion’ of different acrocentrics during meiotic prophase I (Figure 5), in mitotic metaphases, we detected a single association in most cells, which were similar to the ‘fusion’ type. In total, we checked 693 cells: 532 cells had normal karyotype, 32 cells demonstrated chromosomal associations (5.6%), and 126 were aberrant ones (micronuclei, polyploid cells, disturbed anaphases, chromothripsis, etc.). C-banding exposed small blocks of pericentromeric heterochromatin and several intercalary blocks (for two pairs of acrocentrics) (Figure S1c). Small blocks of C-heterochromatin were visible in associated chromosomes, but such blocks were not always merged.

## 4. Discussion

In this work, we describe the variety of chromosomal interactions in male meiosis of *E. alaicus*. Immunocytochemical staining of spermatocytes demonstrated at least four contact types for non-homologous acrocentrics in meiotic prophase I. Starting from clustering inside the heterochromatic cloud, chromosomes demonstrate touching by the elongated axial element of the short arms, then tether by SYCP3 filaments and complete the process when tightly adjacent to each other. As a result, two acrocentrics likely represents a single bivalent with two centromeric regions.

Recently, the first report on connections between non-homologous chromosomes and SC structures was demonstrated by investigating meiosis in CD-1 male mice after irradiation [43]. The authors of this work stressed that the formation of chromosome bridges between non-homologous chromosomes differed from normal endogenous DSB interactions, which did not require connections between axial elements of homologous chromosomes. The wild ancestors of CD-1 mice were bred in a Swiss laboratory in the 1920s. Genomic studies revealed that CD-1 was mostly derived from *M. domesticus* [63], a species with enormous chromosome variability [64]. In this species, specific major and minor satDNA tandem repeats, which are oriented head-to-tail at centromeres [65], may facilitate fusion of mono-armed chromosomes, and build up bi-armed ones. Repeat polarity occurs in telocentric and Rb chromosomes, therefore it was assumed that tandem repetitive satDNA in *M. domesticus* may have been a universal pattern of chromosomal evolution. Numerous Rbs, characteristic for the species, and presumed random associations of bivalents in the wild type, *M. domesticus* (2n = 40) spermatocytes, exemplified a wide spectrum of fusions due to the universal structure of pericentromeric satDNA in this species.

We did not analyze pericentromeric heterochromatin in *Ellobius*, although the numerous associations (at ‘proximity’ type) were similar to *M. domesticus* [43]. Pericentromeric heterochromatin evidently participates in chromosome contacts and fusions. A prelude at the zygotene (Figure S3) may be assigned as an example. Formation of Rbs may depend on telomere changes, especially shortening and inactivation [38]. Recently, the study of wild house mice demonstrated that telomere shortening and the number of critically short telomeres are likely to result in Rb formation [66]. Earlier, we did not detect the telomeric sequences in centromeric regions of numerous Rbs, including Rb(2.11), in *E. tancrei* and *E. talpinus* hybrids [49]. The lack of signal may be explained by elimination as well as inactivation of telomere fragments. The study should be continued in *E. alaicus*.

Contrary to mouse experiments [43], where chromosome contacts were discovered after gamma radiation, *E. alaicus* came from intact natural habitats, close to terra typica of the species. Numerous and various chromosomal contacts, the enormous number of cells with such chromosome ’dance’ in all studied males demonstrated an internal background for evolutionary changes. In natural *E. alaicus* populations, we detected [50] four different Rb translocations Rb(2.11), Rb(1.3), Rb(4.9), Rb(3.10) in distinct combinations, with 2n from 52 to 48. A small number of chromosomal associations in mitotic metaphases, which we demonstrated now for animals with a single pair of Rb(2.11), indirectly confirmed the leading role of changes in the meiotic prophase I. We revealed differences in mitotic cell divisions for fibroblast and chondrocyte cultures. Cell culture often demonstrates unstable karyotype structures and distinct abnormality types and frequencies [67,68]. This assumption was our rationale for comparing data on chromosome numbers and behaviors using bone marrow slides, and two different cell culture approaches. Fibroblasts mostly retained 2n = 52, and numerous polyploid cells. Chondrocyte cultures appeared to be more fragile and demonstrated distinct changes as micronuclei, disturbed anaphases, massive chromosome changes, and associations (Figures S1 and S2). The evolutionary significance of such mutations is incredible if such events appear in the germline [32,69]. The picture of chromosomal re-assemblage (Figure S1) may be an example of chromothripsis, which combines chromosome shattering and fusion [70].

In mitotic cells, we observed two centromeres and two blocks of heterochromatin in the association of two small acrocentrics (Figure S1c,e). Previously, in the pachytene, we observed a small metacentric with two centromeres in all studied animals from natural *E. tancrei* populations, 2n = 34 (as in Figure S8). *E. tancrei* demonstrates a wide spectrum of homologous and non-homologous Robertsonian metacentrics in natural populations [51]. We suggest that dicentric chromosome formation in natural populations of this species, confirms the ’contact first in meiosis’ model.

Although we had no meiotic data to prove double-stranded DNA breaks and chromosome reassembling, we demonstrated that the ‘contact-first’ scheme may be applicable to meiotic translocation mechanisms. Another question—the fate of small arms of acrocentric chromosomes, which are apparently kept in fused chromosomes (Figure 4e,f and Figure 5c)—is closely related to the role and evolution of the centromere. As previously mentioned, we supposed the neocentromere origin was a crucial step for *E. tancrei* and *E. alaicus* genome evolution. In cases of ‘fused’ chromosomes of *E. alaicus,* we revealed two distinct centromere signals using CREST immunostaining. The question of the further fate of two centromeres and chromosome fragments between them is now open, and the study should be continued.

Considering that mitosis and meiosis data are consistent, we can argue for the essential role of heterochromatin. In mouse spermatocytes, heterochromatin preserves the association of homologous centromeres and promotes faithful chromosome segregation in meiosis I [71]. Heterochromatin, in maintaining genome stability [72], may be a focal point for changes. We demonstrated that a ‘proximity’ to fuse correlated with the heterochromatin affinity of non-homologous chromosomes. Chromosomes were located around the H3K9me3-heterochromatin cloud (Figure 6f,i), similar to telocentric associations in mice [44]. Clustering of heterochromatic regions inside the H3K9me3 clouds was demonstrated for different sets of acrocentrics in *Mus musculus domesticus* either for the wild type 2n = 40 or for different forms with Rbs [44,73,74]. This clustering could be the background of the translocations. However, no data on contacting chromosomes were published, except the gamma radiation treatment caused the formation of bridges between chromosomes [43]. Earlier, we described the participation of heterochromatin of short chromosome arms in the formation of SC chains in mole voles heterozygous for multiple Rbs [75].

High natural variability along with the rapid fixation of new translocations and specific contacts of non-homologous chromosomes in meiosis, which we demonstrated for *E. alaicus*, built a promising background to change the model for the mechanism of translocations from the ‘contact first’ to the ‘contact first in meiosis’.

The evolutionary significance of meiosis as a tool to increase recombinational diversity is balanced by its function as a mechanism for purifying selection [76]. Meiotic checkpoints block cell cycle progression in response to defects and preclude abnormal chromosome segregation. Nevertheless, creative meiosis became apparent if we apply the ‘contact-first’ model for meiotic prophase I, as we demonstrated for a unique rodent species, which entered the stage of Robertsonian translocations emergence.

## Supplementary Materials

The following are available online at https://www.mdpi.com/2073-4425/11/4/386/s1, Figure S1: Mitotic chromosomes of *Ellobius alaicus*, Figure S2: Aberrations in mitosis of *Ellobius alaicus*, Figure S3: Zygotene spermatocyte of *E.alaicus*, Figure S4: Different chromosome combinations in pachytene spermatocytes of *E.alaicus*, Figure S5: Different chromosome combinations in pachytene spermatocytes of *E.alaicus*, Figure S6: Location of SYCP3, H3K9me3, CREST and chromatin configuration (DAPI) in zygotene—pachytene stages of prophase I of *E. alaicus* spermatocytes, Figure S7: Location of SYCP3, H3K9me3, CREST, and chromatin configuration (DAPI) in pachytene—diakinesis stages of the prophase I of *E. alaicus* spermatocytes, Figure S8: A pachytene oocyte of *E. tancrei,* 2n = 34, NF = 56.
