# Supplementary material for: Meiotic Chromosome Contacts as a Plausible Prelude for Robertsonian Translocations
Source: Genes (Basel). 2020 Apr 2;11(4):386. doi: 10.3390/genes11040386 (PMC7230836; doi:10.3390/genes11040386)
Supplement: Supplementary file 1 [file genes-11-00386-s001.doc]

**Supplementary materials**

Meiotic Chromosome Contacts as a Plausible Prelude for Robertsonian Translocations

Sergey Matveevsky, Oxana Kolomiets, Aleksey Bogdanov, Elena Alpeeva and Irina Bakloushinskaya


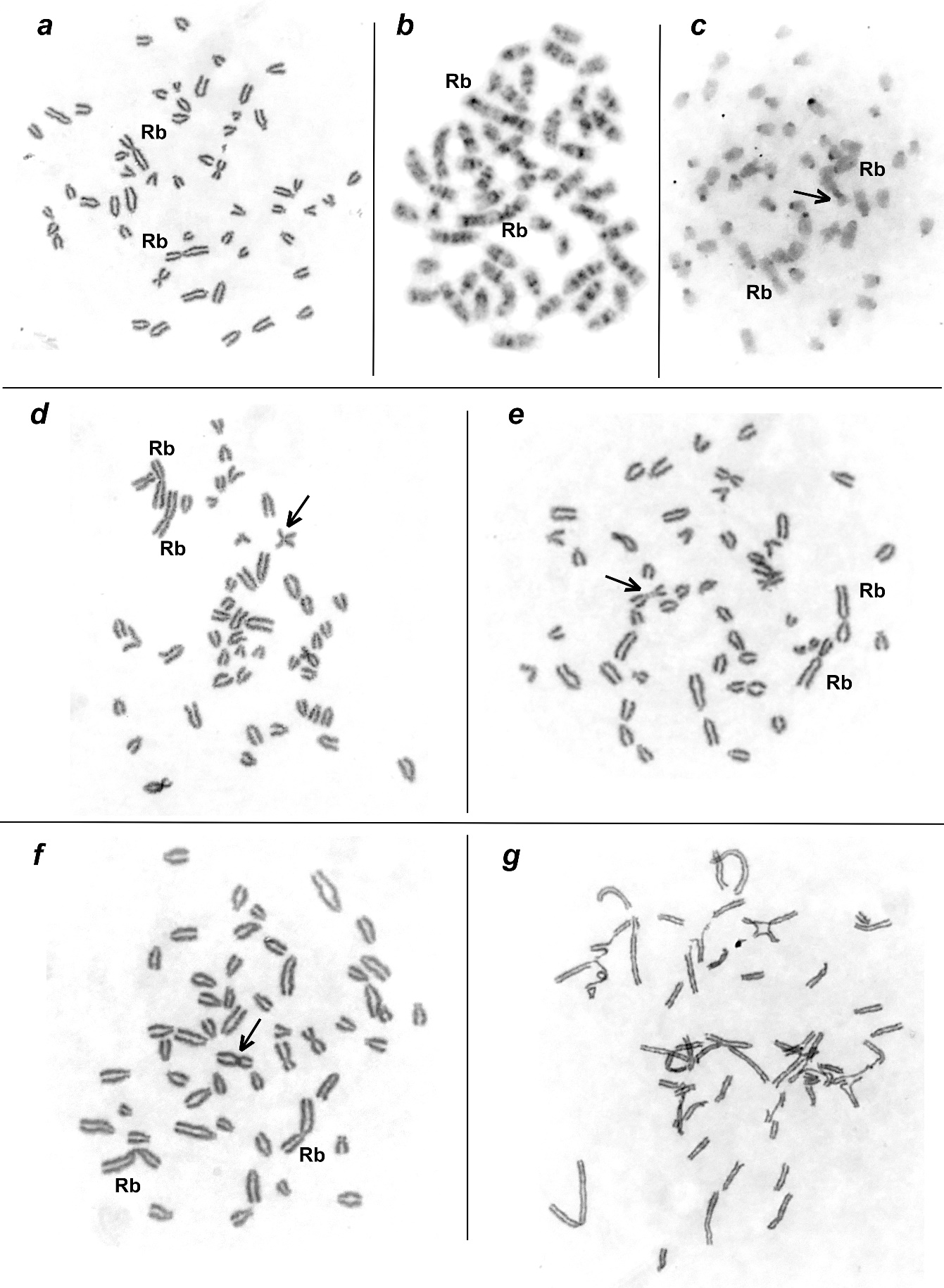


Figure S1. Mitotic chromosomes of *Ellobius alaicus*. (a) routine staining, 2735, female, (b) G-banding, bone marrow, 27352, male (c) C-banding, 27351, female; associations are marked by an arrow, 27351, female (d-f); chromothripsis, 27351, female (g).


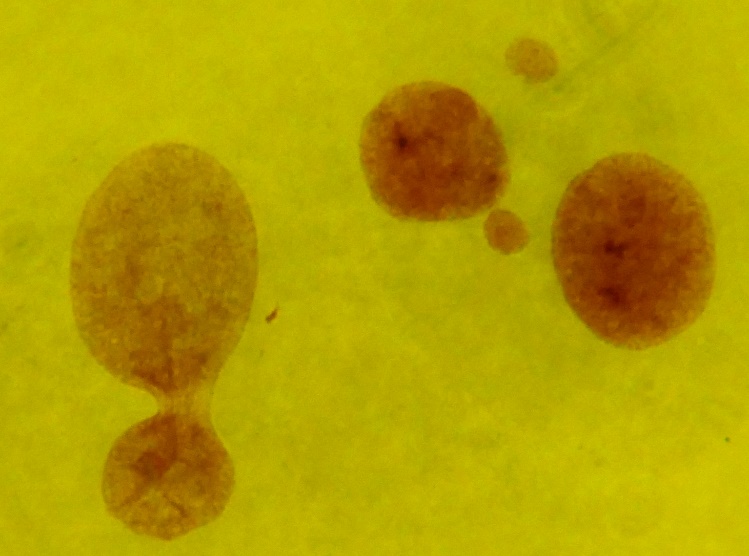


Figure S2. Aberrations in mitosis of *Ellobius alaicus*. 27351, female: an anaphase bridge and micronuclei.


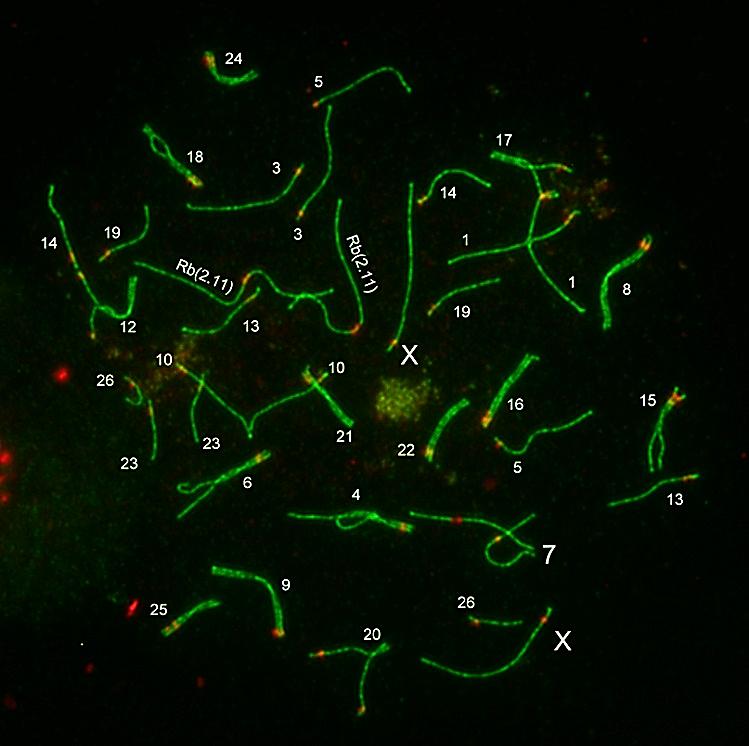


Figure S3. Zygotene spermatocyte of *E.alaicus*. Axial elements were identified using anti-SYCP3 antibodies (green), CREST antibodies for kinetochores.


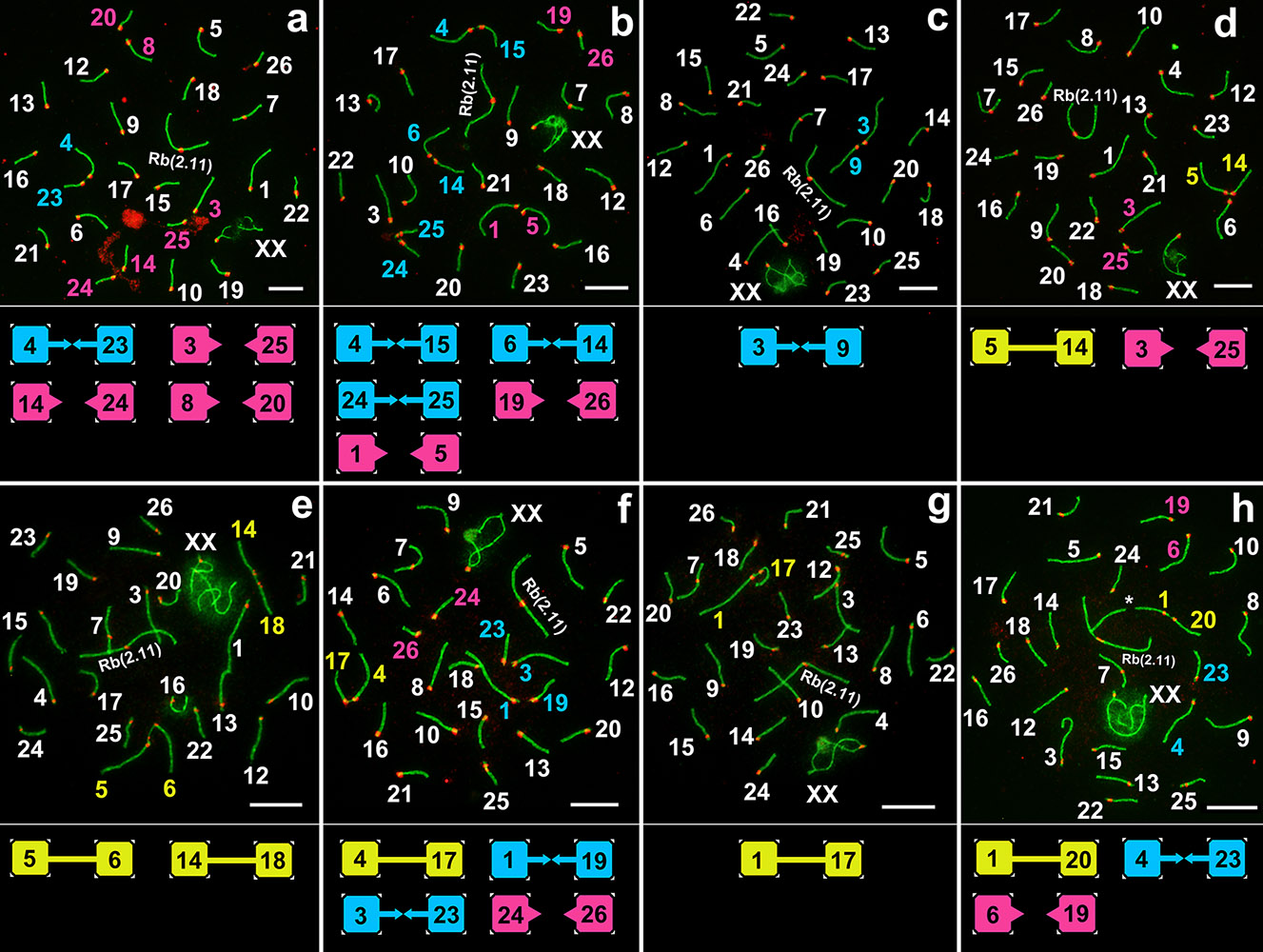


Figure S4. Different chromosome combinations in pachytene spermatocytes of *E.alaicus*. Axial elements were identified using anti-SYCP3 antibodies (green) and anti-CREST for kinetochores (red). Some symbols see in Figure 3. Bar (**a**–**h**) = 5 µm.


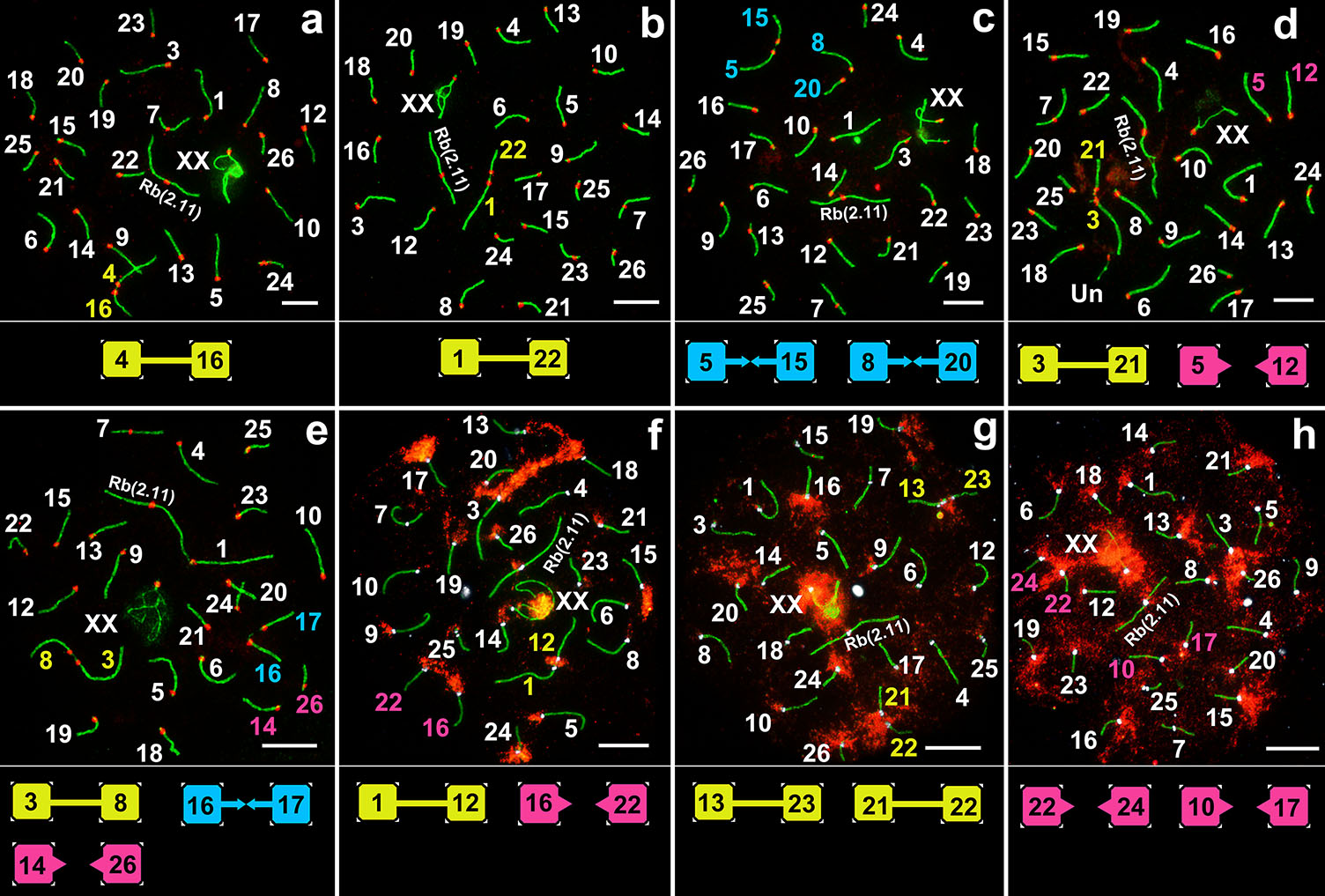


Figure S5. Different chromosome combinations in pachytene spermatocytes of *E.alaicus*. Axial/lateral elements were identified using anti-SYCP3 antibodies (green), anti-CREST for kinetochores (red for a-e and white for f-h) and anti-H3K9me3 as marker of heterochromatin (red for f-h). Symbols as in Figure. 3. Bar (**a**–**e**) = 5 µm.


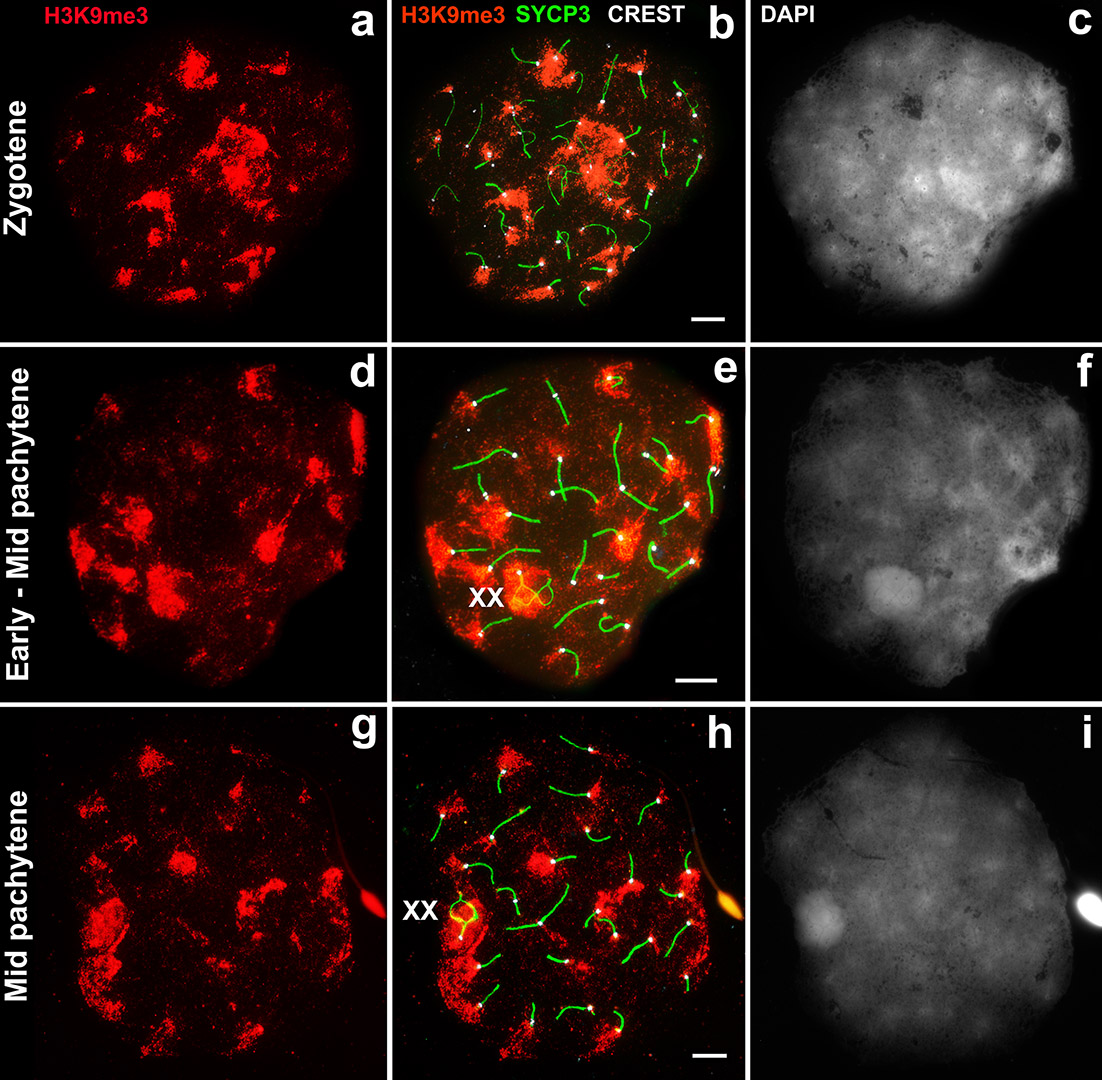


Figure S6. Location of SYCP3 (green), H3K9me3 (red), CREST (white) and chromatin configuration (DAPI, grey) in zygotene – pachytene stages of prophase I of *E. alaicus* spermatocytes. Bar (**a**–**i**) = 5 µm.


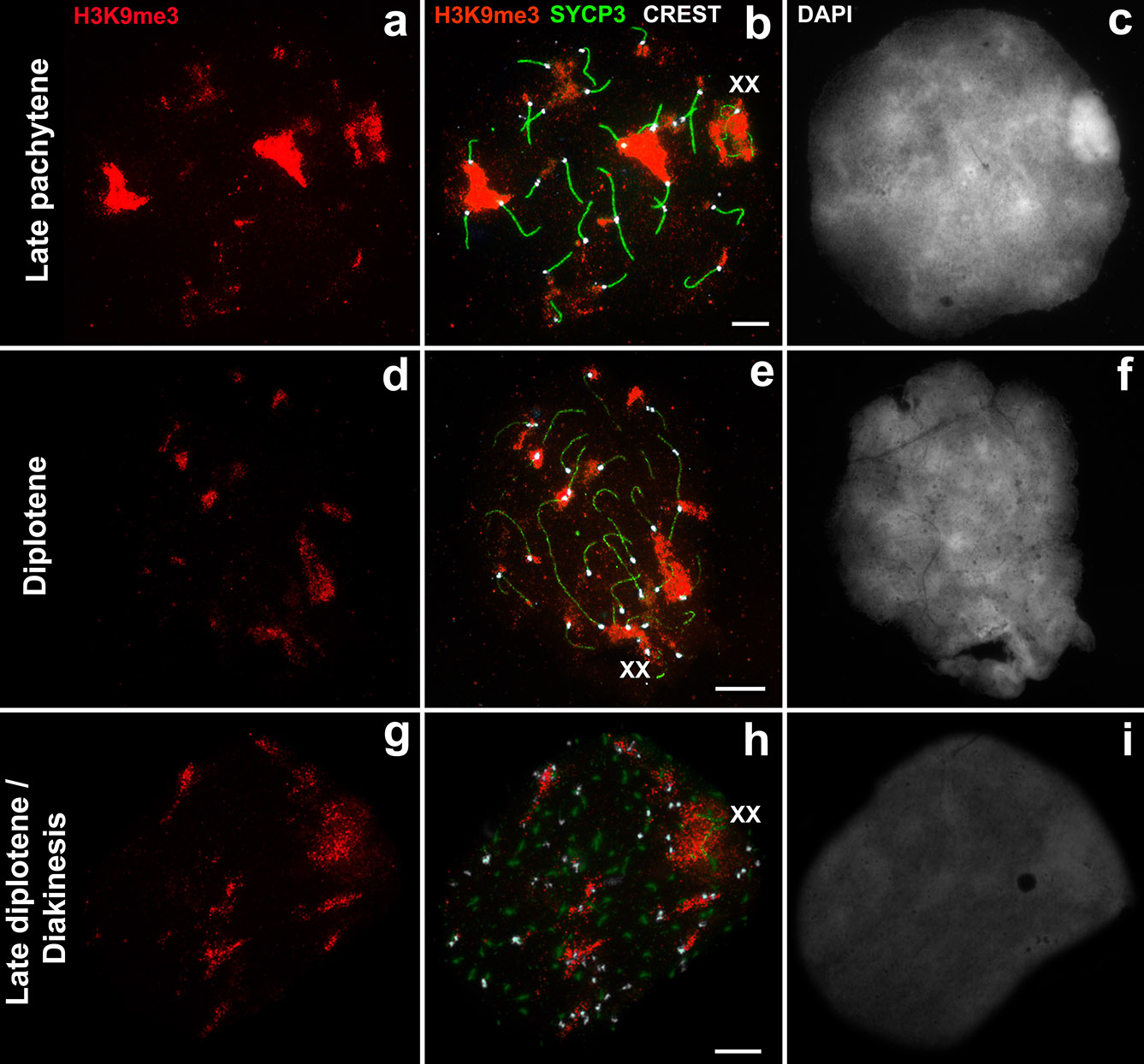


Figure S7. Location of SYCP3 (green), H3K9me3 (red), CREST (white), and chromatin configuration (DAPI, grey) in pachytene – diakinesis stages of the prophase I of *E. alaicus* spermatocytes. Bar (**a**–**i**) = 5 µm.


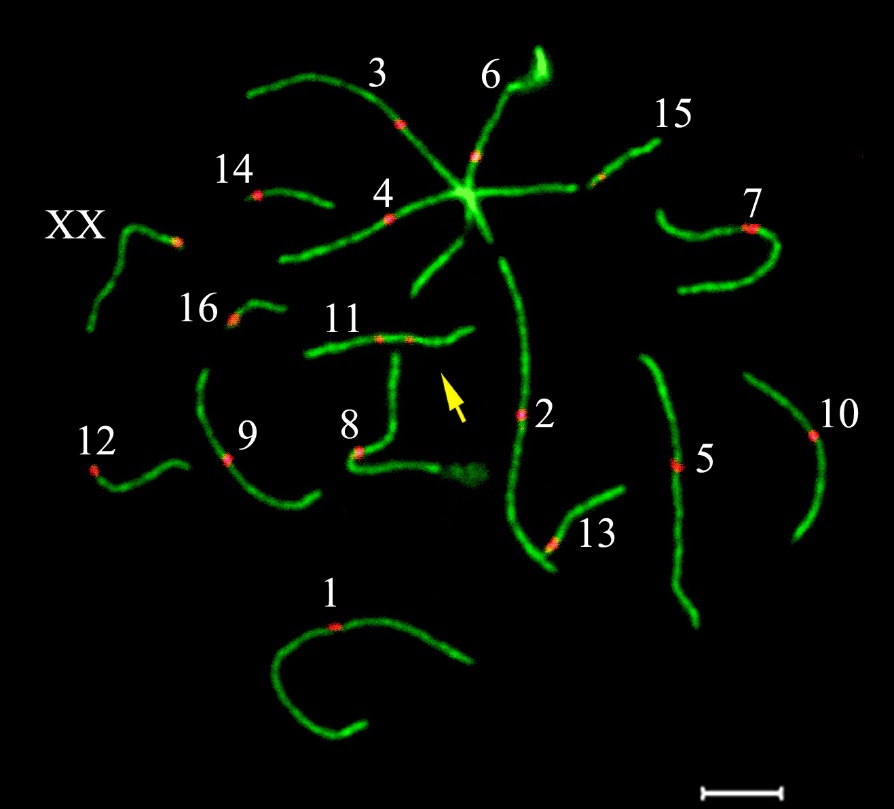


Figure S8. A pachytene oocyte of *E. tancrei,* 2n=34, NF=56. The bivalent #11 with two centromeres is marked by the yellow arrow. Bar = 5 µm.
